# Cloning and expression of genes encoding heat shock proteins in *Liriomyza trifolii* and comparison with two congener leafminer species

**DOI:** 10.1371/journal.pone.0181355

**Published:** 2017-07-20

**Authors:** Ya-Wen Chang, Jing-Yun Chen, Ming-Xing Lu, Yuan Gao, Zi-Hua Tian, Wei-Rong Gong, Chang-Sheng Dong, Yu-Zhou Du

**Affiliations:** 1 School of Horticulture and Plant Protection & Institute of Applied Entomology, Yangzhou University, Yangzhou, China; 2 Laboratory for Prevention and Control of Alien Pests, Suzhou Entry-Exit Inspection and Quarantine Bureau, Suzhou, China; 3 Joint International Research Laboratory of Agriculture and Agri-Product Safety, Yangzhou University, Yangzhou, China; 4 Plant Protection and Quarantine Station of Jiangsu Province, Nanjing, China; 5 Agricultural Technology Extension Service Center of Guangling District, Yangzhou, China; Chinese Academy of Agricultural Sciences Institute of Plant Protection, CHINA

## Abstract

The polyphagous agromyzid fly, *Liriomyza trifolii*, is a significant and important insect pest of ornamental and vegetable crops worldwide. The adaptation of insects to different environments is facilitated by heat shock proteins (HSPs), which play an important role in acclimation to thermal stress. In this study, we cloned and characterized five HSP-encoding genes of *L*. *trifolii* (*Lthsp20*, *Lthsp40*, *Lthsp60*, *Lthsp70*, and *Lthsp90*) and monitored their expression under different thermal stresses using real-time quantitative PCR. Pupae of *L*. *trifolii* were exposed to 19 different temperatures ranging from -20 to 45°C. The results revealed that *Lthsp20*, *Lthsp40*, *Lthsp70* and *Lthsp90* were significantly upregulated in response to both heat and cold stress, while *Lthsp60* was induced only by heat temperatures. The temperatures of the onset (*T*_on_) and maximal (*T*_max_) expression of the five *Lthsps* were also determined and compared with published *T*_on_ and *T*_max_ values of homologous genes in *L*. *sativae* and *L*. *huidobrensis*. Although *L*. *trifolii* occurs primarily in southern China, it has cold tolerance comparable with the other two *Liriomyza* species. Based on the heat shock proteins expression patterns, *L*. *trifolii* has the capacity to tolerate extreme temperatures and the potential to disseminate to northern regions of China.

## Introduction

*Liriomyza trifolii* is an economically important invasive insect pest in China [1]. It was initially discovered in Guangdong in 2005 [2] and has since proliferated throughout the southern region of China [3]. Both larvae and adults of *L*. *trifolii* can cause damage to crop plants. The larvae mine tunnels in the leaf tissues, and female adults puncture the leaf tissues for oviposition. Both activities can reduce photosynthesis and increase leaf drop, resulting in lower crop quality and yield [4–5]. In recent years, *L*. *trifolii* has spread rapidly throughout the country, causing significant damage to various vegetable and horticultural crops [6–8].

Insects are poikilothermic organisms, and their physiological activities can be greatly affected by temperatures [9]. The tolerance of insects to temperature stress is a definitive factor in their survival [10–11]. Multiple studies have shown that insect tolerance to thermal stress is multifactorial and has genetic, physiological, and biochemical components [12–17]. Insects exposed to temperature stress may exhibit alterations in behavior, such as seeking shelter. Additionally, changes in morphology, life history and physiological characteristics, which include changes in membrane fluidity, the accumulation of carbohydrate alcohols, and the generation of heat shock proteins (HSPs) and antioxidant enzymes are also shown to effect in tolerate extreme temperatures [18–19]. Alternations of *hsps* expression in insects as affected by temperature stress has been widely studied and is one of the best predictors of insect tolerance to temperature stress [20].

Insect HSPs are divided into several families based on molecular weight and homology, including HSP90, HSP70, HSP60, HSP40 and small heat shock proteins (sHSPs) [21–23]. In addition to increasing heat tolerance and protecting organisms from thermal injury, HSPs function as molecular chaperones to promote proper protein folding and prevent the aggregation of denatured proteins [20, 24–26].

Previous studies have examined the response of *hsp*90, *hsp*70, *hsp*60, *hsp*40 and *hsp*20 to temperature stress in *L*. *sativae* and *L*. *huidobrensis* [27], and *hsp90* and *hsp70* in *L*. *trifolii* [28–29]. However, the expression profiles of *hsp*60, *hsp*40 and *hsp*20 in *L*. *trifolii* during temperature stress has not yet been investigated. In this study, we characterized the five *hsps* in *L*. *trifolii*, *hsp*90, *hsp*70, *hsp*60, *hsp*40 and *hsp*20 to better understand *hsp* expression in response to both high and low temperature stress. In addition, we also compared the expression of *hsps* in *L*. *trifolii* with the homologous genes in *L*. *sativae* and *L*. *huidobrensis*, which provides insights into the competition between *Liriomyza* spp. and the distribution and dissemination of leaf mining insects in response to temperature.

## Materials and methods

### Study insect

*L*. *trifolii* were originally collected on celery in Yangzhou (32.39°N, 119.42°E) in 2010 and reared on beans in the laboratory at 26°C with a 16:8 h (L: D) photoperiod as described by Chen & Kang [30]. Beans (*Vigna unguiculata*) were seeded at the rate of 5–6 plants per pot (12 cm in diameter) and moved into cages (40×40×65cm) for insect feeding when plants had five to six true leaves. About 150 adults were reared per cage and the larvae inside the tunneling leaves were collected in plastic bags until pupation. The pupae were collected in glass tubes and no field populations were added during experimental period. No specific permissions were required for these activities and the field studies did not involve endangered or protected species.

### Temperature treatments

Two-day-old pupae (n = 30) were collected and placed in small glass tubes. The glass tubes along with the pupae were placed into a temperature controller (DC-3010, Ningbo, China) and exposed for 1 h at low temperatures of -20, -17.5, -15–12.5, -10, and -7.5°C; moderate temperatures of -5, -2.5, 0.0, 2.5, 27.5, and 30°C; and high temperatures of 32.5, 35, 37.5, 40, 42.5, and 45°C. The control group consisted of the pupae maintained at 25°C. After exposure to thermal treatments, pupae were allowed to recover at 25°C for 1 h, frozen in liquid nitrogen, and stored at -70°C. Each treatment was repeated four times.

### RNA isolation and cloning experiments

Total RNA was extracted from *L*. *trifolii* using the SV Total RNA isolation system (Promega, USA). The integrity and purity of RNA was determined by agarose gel electrophoresis and spectrophotometry (Eppendorf Bio Photometer plus, Germany). Total RNA (1 μg) was transcribed into cDNA using oligo (dT) primers. Degenerate primers (Table 1) were used to amplify partial segments of the five *hsps*, and then 5′ and 3′ RACE were utilized to obtain the full-length cDNAs as recommended by the manufacturer (SMART RACE cDNA Amplification Kit, Clontech, USA).

**Table 1:** Primers used in the cDNA cloning and real-time quantitative PCR.

Gene*		Primer sequences(5’→3’)	Fragment length (bp)	
Primers for cDNA cloning				
*hsp20*	F	ATGTDCAACARTTYGCYCC		187
	R	ACGCCGTCDGADGAMARTTG		
	5’	CCTCCACTACCACATAGTTGTCCACCA		451
	3’	ACGCTACCGTCTACCTAAGGGTGTCA		405
*hsp40*	F	GCGGTGGYGCYTTYCGTT		338
	R	CACTGCCATCCCGTTTGA		
	5’	GGAAATAGTTTTGACCACCTGGGCTG		609
	3’	CCTTCCCCAAAGAGGGTGACCAATC		510
*hsp60*	F	CAAGTRGCCACMATCTCA		931
	R	TTGCCGTAYTCRCCCTTM		
	5’	TTCCACCTTAGCTCCCTTGGAGGAA		948
	3’	GCCGCATAGAGAAGGTATTGTGCCC		621
*hsp70*	F	TCAAACGCAAATACAAGAAGGAT		731
	R	TACATTCAGTATGCCGTTAGCGT		
	5’	GACAGATTCAGGCTCTTGCCACAG		927
	3’	ACTTGGACGCTAACGGCATACTGA		683
*hsp90*	F	CCAAGTCTGGTACTAAGGCA		776
	R	CAAATCTTCACAGTTGTCCATGA		
	5’	TATGTATGGGAATCATCAGCTGG		618
	3’	GTGCGCCGTGTGTTCATCATGGA		1441
*β-actin*	F	TACCAACTGGGATGATATGGAA		322
	R	TCGACCAGCCAAGTCCAAACGC		
Primers for qRT-PCR				
*hsp20*	F	GAAATCAATGTGAAAGTGGTGGA		175
	R	GAACCTTCAACAAGCCATCAGAT		
*hsp40*	F	AAAGTCTCACTCAAGCAAGCATT		127
	R	GTCCAGTTATGCGTTTGACAGTT		
*hsp60*	F	AAATAGTGCGTCGTTCATTGCGT		99
	R	CGGATTGTGTTTCAACTTTAGCC		
*hsp70*	F	GTCGCATACCAAGCAAACAAAC		198
	R	CGTTAGCGTCCAAGTCAAATGT		
*hsp90*	F	CAAAACTAAACCCATCTGGACACG		158
	R	GCACAAACAAAAGAGCACGGA		
*β-actin*	F	TTGTATTGGACTCTGGTGACGG		73
	R	GATAGCGTGAGGCAAAGCATAA		

* F, forward; R, reverse; 5’, 5’ RACE primer; 3’, 3’ RACE primer.

### Quantitative real-time reverse transcriptase PCR (qRT-PCR)

RNA (0.5 μg) was reverse-transcribed into first-strand cDNA using the Bio-Rad iScript™ cDNA Synthesis Kit (Bio-Rad, CA, USA). Reactions were conducted in a 20 μl reaction volume consisting of 10 μl Bio-Rad iTaq™ Universal SYBR^®^ Green Supermix (2×), 1 μl of each gene-specific primer (10 μM) (Table 1), 2 μl of each cDNA template, and 6 μl of ddH_2_O. Real-time PCR were performed using an Applied Biosystems 7500 real-time PCR system (Thermo Fisher Scientific, USA) under the following conditions: 3 min at 95°C, 40 cycles of denaturation at 95°C for 30 s, and annealing at the *T*_*m*_ of primer pairs (Table 1) for 30 s. Each treatment contained four replications, and each reaction was run in triplicate. *β-actin* was cloned from *L*. *trifolii* (GenBank accession no: KY231150) and used as a reference gene.

### Sequence alignment and data analysis

Full-length cDNAs sequences of the five *Lthsps* were used as queries to search for other insect *hsps* using the BLAST programs available at the NCBI website (http://www.ncbi.nlm.gov/BLAST/). Sequence alignments were conducted using Clustal X software [31]. Open reading frames (ORFs) were identified using ORF Finder (https://www.ncbi.nlm.nih.gov/orffinder/). Sequence analysis tools of the ExPASy Molecular Biology Server (Swiss Institute of Bioinformatics, Switzerland) were used to analyze the deduced *hsps* sequences.

The 2^−ΔΔ^Ct method was used to evaluate fold changes in mRNA expression levels [32]. Geometric means of the reference genes were utilized to normalize expression under different experimental conditions. One-way ANOVA was used to detect significant differences in mRNA levels among treatments, followed by Tukey’s multiple comparison (*P*<0.05) in SPSS v. 16.0 (SPSS, Chicago, IL, USA). For ANOVA tests, original data were log-transformed for homogeneity of variances. The temperature where expression was significantly higher than that at 25°C was designated as the onset temperature (*T*_on_), whereas the temperature where expression was significantly higher than that of other temperatures was denoted as *T*_max_.

## Results

### Cloning and characterization of five *hsps* from *L*. *trifolii*

The five *hsps* cloned from *L*. *trifolii* were designated as *Lthsp20*, *Lthsp40*, *Lthsp60*, *Lthsp70*, and *Lthsp90*, respectively, and were deposited in GenBank with accession nos. KY231145, KY231146, KY231147, KY231148 and KY231149, respectively. The full-length cDNAs of *Lthsp20*, *Lthsp40*, *Lthsp60*, *Lthsp70*, and *Lthsp90* were 911, 1490, 2065, 2293, and 2639bp, respectively. The sequence information of the five *Lthsps* and its predicted amino acids were detailed in Table 2.

**Table 2:** The sequence information of the five *Lthsp* genes and its predicted amino acids.

Gene	cDNA length (bp)	5’UTR length (bp)	3’UTR length (bp)	ORF length (bp)	molecular weight (kDa)	isoelectric point	Accession number
*hsp20*	911	132	218	561	21.23	6.38	KY231145
*hsp40*	1490	289	181	1020	37.92	8.94	KY231146
*hsp60*	2065	169	177	1719	60.96	5.73	KY231147
*hsp70*	2293	184	186	1923	70.43	5.43	KY231148
*hsp90*	2639	132	362	2145	81.63	4.96	KY231149

**Fig 1:**
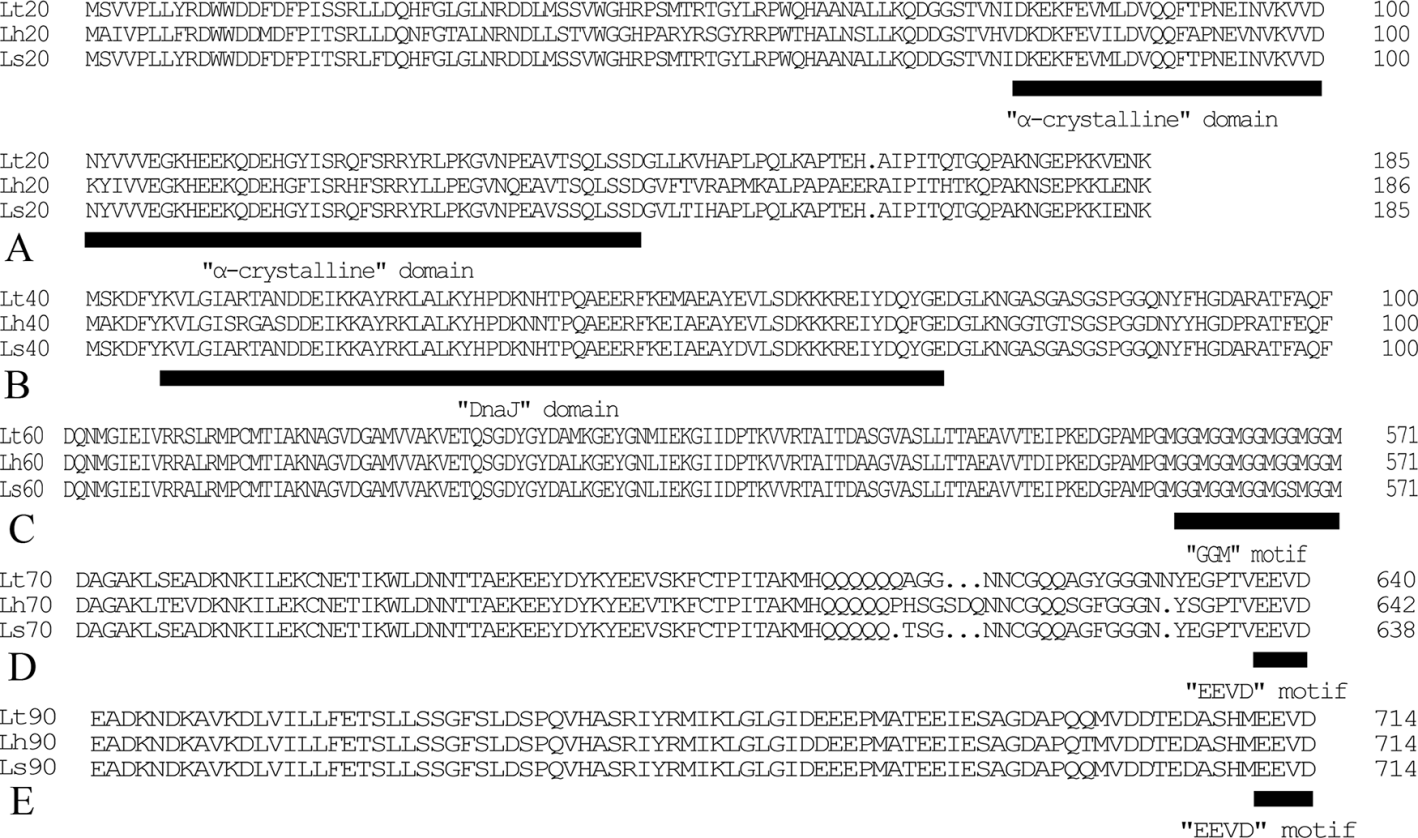
Salient features of five genes encoding HSPs in *Liriomyza* spp. The amino acid sequences of the deduced protein products of *hsp20* (A), *hsp40* (B), *hsp60* (C), *hsp70* (D) and *hsp90* (E) were aligned and conserved motifs or domains are indicated. Dots (.) indicate alignment. Abbreviations: Lt20, *L*. *trifolii hsp20*; Lh20, *L*. *huidobrensis hsp20* (DQ452370.1); Ls20, *L*. *sativae hsp20* (DQ452371.1); Lt40, *L*. *trifolii hsp40*; Lh40, *L*. *huidobrensis hsp40* (DQ452364.1); Ls40, *L*. *sativae hsp40* (DQ452365.1); Lt60, *L*. *trifolii hsp60*; Lh60, *L*. *huidobrensis hsp60* (AY845949.2); Ls60, *L*. *sativae hsp60* (AY851366.2); Lt70, *L*. *trifolii hsp70*; Lh70, *L*. *huidobrensis hsp70* (AY842476.2); Ls70, *L*. *sativae hsp70* (AY842477.2); Lt90, *L*. *trifolii hsp90*; Lh90, *L*. *huidobrensis hsp90* (AY851367.2); and Ls90, *L*. *sativae hsp90* (AY851368.2).

The alignment of *Lt*HSP20 with sHSPs from *L*. *sativae* and *L*. *huidobrensis* revealed a conserved region in the middle, which constitutes an α-crystalline domain (Fig 1A). The N- and C-terminal ends of the predicted proteins were highly variable in the three *Liriomyza* spp. HSP20s from *L*. *trifolii* and *L*. *sativae* showed 95.70% amino acid identity, whereas HSP20 orthologs in *L*. *trifolii* and *L*. *huidobrensis* only showed 72.73% identity. *Lt*HSP40 showed 96.76 and 88.53% amino acid identity with orthologous proteins in *L*. *sativae* and *L*. *huidobrensis*, respectively. The N-terminal 65 amino acids (positions 4–68), which constitute the most conserved region of HSP40, comprise the DnaJ domain (Fig 1B). *Lt*HSP60 showed a high degree of identity to related proteins in *L*. *sativae* and *L*. *huidobrensis* (95.63 and 95.98% identity, respectively). *Lt*HSP60 contained a conserved GGM motif at the C-terminal end (Fig 1C). Multiple ATP/Mg^2+^ binding sites were distributed throughout the predicted protein product in *L*. *trifolii*, which were consistent with the structure of HSP60s in the two other *Liriomyza* spp. Amino acid alignments revealed that *Lt*HSP70 was closely related to analogous proteins in *L*. *sativae* and *L*. *huidobrensis*, which showed 99.06 and 95.96% amino acid identity, respectively. Similarly, HSP90 in *L*. *trifolii* showed a high degree of identity relative to that in *L*. *huidobrensis* and *L*. *sativae* (97.34 and 99.30%, respectively). Conserved EEVD motifs were identified in the C-terminal ends of *Lt*HSP90 and *Lt*HSP70 (Fig 1D and 1E).

### Expression of *Lthsps* at different temperatures

**Fig 2:**
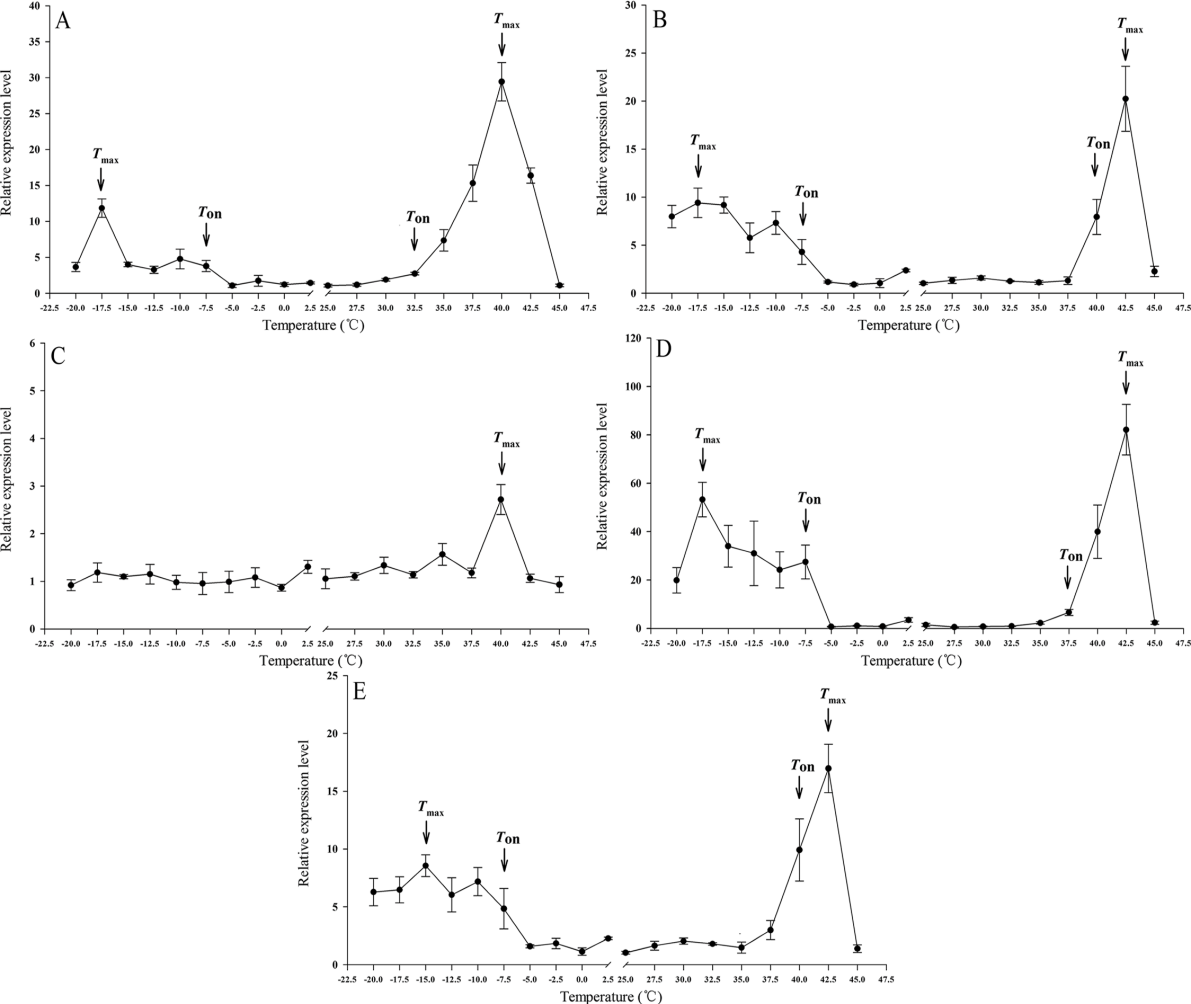
The mRNA expression profiles of genes encoding five HSPs in *L*. *trifolii*. Panels: (A) *hsp20*; (B) *hsp40*; (C) *hsp60*; (D) *hsp70*; and (E) *hsp90*. The first temperature where expression was significantly higher than that the control (25°C) was described as the onset temperature (*T*_on_) or the *hsp*, and the temperature at which the expression level was significantly higher than expression at other temperatures was denoted as *T*_max_. *T*_on_ and *T*_max_ are marked by arrows (→), and the notable temperature shifts of *T*_on_ and *T*_max_ are indicated on the curves. The relative level of *hsp* expression represented the fold increase as compared with the expression in controls. The data were denoted as mean ± SE.

The expression of *Lthsps* in response to temperature stress (-20 to 45°C) was examined using qRT-PCR. The results showed that the *Lthsp20*, *40*, *70*, and *90* were all significantly upregulated in response to both cold and heat stress (cold stress: *F*_6, 21_ ≥ 9.818, *P* < 0.001; heat stress: *F*_6, 21_ ≥ 6.631, *P* < 0.001). The *Lthsp60* also showed significant differences (*F*_6, 21_ = 6.994, *P* < 0.001) under heat stress, with mRNA levels increased by 2.72-fold after 1 h at 40°C, while it did not show different responses to cold stress (*F*_6, 21_ = 0.412, *P* = 0.863) (Fig 2C). The expression of the five *Lthsps* was inhibited when temperatures were lower than -17.5°C or higher than 42.5°C (Fig 2). The *Lthsps* showed different expression patterns in response to temperature. *Lthsp20*, *40*, *70*, and *90* showed a dramatic increase in expression in response to heat stress with mRNA expression increased by 29.43-, 20.24-, 82.15-, and 16.97-fold, respectively, after 1 h at 40°C or 42.5°C (Fig 2A, 2B, 2D and 2E). However, the five *Lthsps* were not induced by relatively mild temperatures (-5°C, -2.5°C, 0°C, 2.5°C, 27.5°C, and 30°C) (*F*_6, 21 ≤_ 2.491, *P ≥* 0.056) (Fig 2).

The relative mRNA levels of *Lthsps* were compared and the onset (*T*_on_) and maximal (*T*_max_) temperature values were identified. Under cold temperature stress, the *T*_on_ values of these five *Lthsps* were all in -7.5°C and the *T*_max_ values were -17.5°C, except for *Lthsp90*, which peaked at -15°C. In response to heat stress, the *T*_on_ values were 32.5°C for *Lthsp20*, 40°C for *Lthsp40* and *Lthsp90*, and 37.5°C for *Lthsp70*. The *T*_max_ was 40°C for *Lthsp60* and *Lthsp20* and 42.5°C for the other three *Lthsps* (Fig 2).

### Interspecific differences in *hsps*

**Fig 3:**
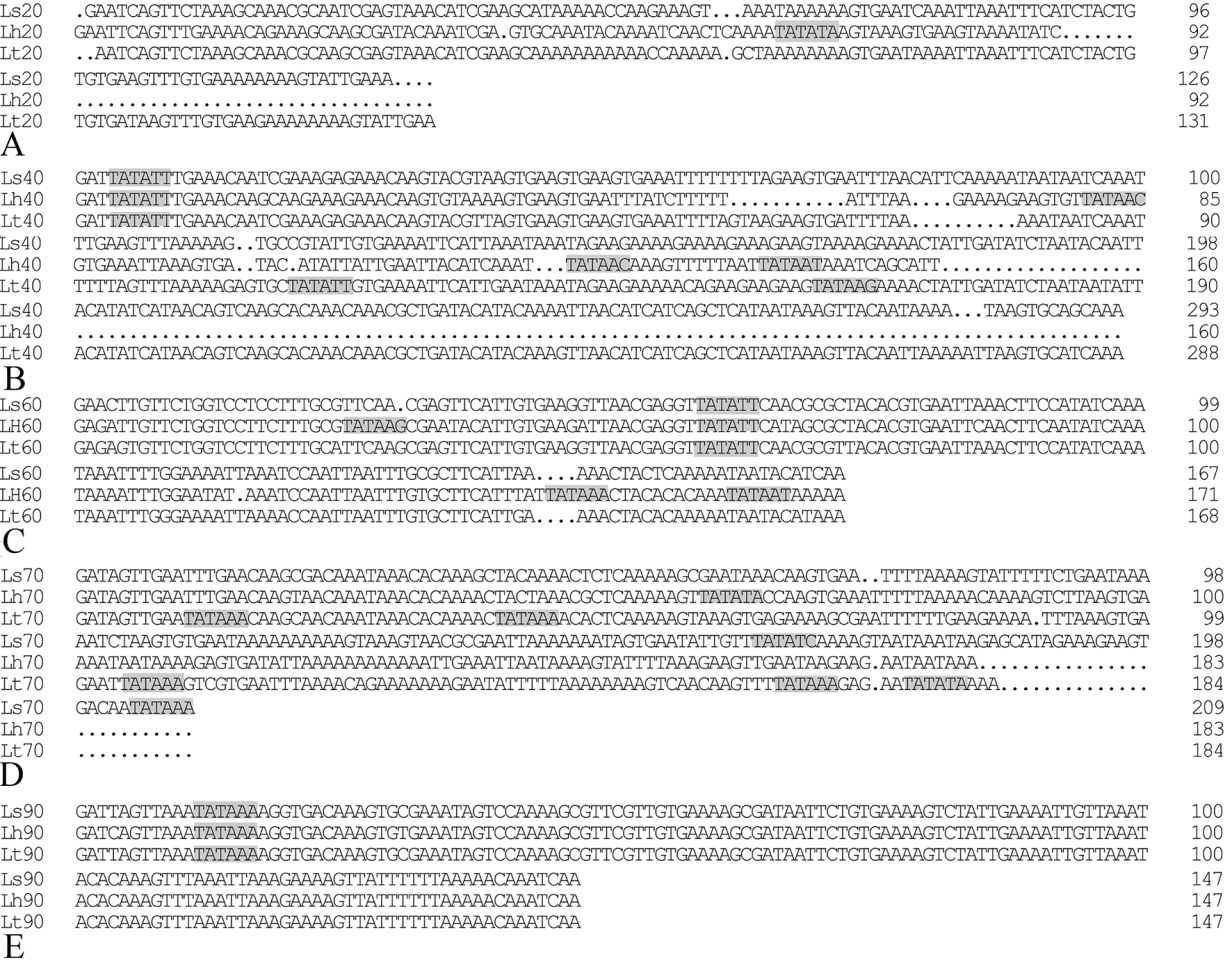
Alignment of 5’UTRs of *Liriomyza hsp* genes. The TATA-box-like elements are indicated by shading and the dots indicate alignment. Abbreviations are identical to those in Fig 1. Panels: (A) *hsp20*; (B) *hsp40*; (C) *hsp60*; (D) *hsp70*; and (E) *hsp90*.

A total of ten TATA-box-like regulatory elements were identified in the 5’ untranslated regions (5’UTRs) of the five *Lthsps*. In comparison, five and eleven TATA-box-like elements were identified in the 5’UTRs of *hsps* in *L*. *sativae* and *L*. *huidobrensis*, respectively (Fig 3). *Liriomyza huidobrensis* contained a single TATA-box-like element in the 5’UTR of *hsp20*, which was not present in the other two *Liriomyza* spp. (Fig 3A). The 5’UTR of *hsp40* contained three, four, and one TATA-box in *L*. *trifolii*, *L*. *huidobrensis*, and *L*. *sativae*, respectively (Fig 3B). In *hsp60*, *L*. *trifolii* and *L*. *sativae* possessed a single TATA-box-like element but *L*. *huidobrensis* had four (Fig 3C). Five TATA-box-like elements were found in *Lthsp70*, whereas *L*. *huidobrensis* and *L*. *sativae* contained one and two, respectively (Fig 3D). All three leafminers contained a single TATA-box-like element in the 5’ UTR of *hsp90* (Fig 3E).

Under cold stress, the *T*_on_ and *T*_max_ values of the five *hsps* in *L*. *trifolii* and *L*. *huidobrensis* were 2.5 to 7.5°C lower than that in *L*. *sativae* (Table 3). The *T*_on_ and *T*_max_ values were generally similar among the three leafminer species when exposed to heat stress, with the exception of *T*_on_ for *hsp90*, which varied from 30 to 40°C in the three leafminer species (Table 3).

**Table 3:** Interspecific differences in *T*_on_ and *T*_max_ between three species of *Liriomyza* leafminers.

Gene	*L*. *trifolii*				*L*. huidobrensis*				*L*. sativae*			
	heat		cold		heat		cold		heat		cold	
	*T*_on_	*T*_max_	*T*_on_	*T*_max_	*T*_on_	*T*_max_	*T*_on_	*T*_max_	*T*_on_	*T*_max_	*T*_on_	*T*_max_
*hsp20*	32.5°C	40°C	-7.5°C	-17.5°C	30°C	40°C	-7.5°C	-17.5°C	32.5°C	42.5°C	0°C	-12.5°C
*hsp40*	40°C	42.5°C	-7.5°C	-17.5°C	37.5°C	40°C	-5°C	-12.5°C	40°C	42.5°C	-2.5°C	-10°C
*hsp60*	-	40°C	-	-	-	37.5°C	-	-	-	42.5°C	-	-
*hsp70*	37.5°C	42.5°C	-7.5°C	-17.5°C	32.5°C	40°C	-7.5°C	-17.5°C	35°C	42.5°C	-2.5°C	-10°C
*hsp90*	40°C	42.5°C	-7.5°C	-15°C	30°C	40°C	-5°C	-15°C	40°C	42.5°C	-2.5°C	-12.5°C

*Data for *L*. *huidobrensis* and *L*. *sativae* were obtained from Huang and Kang [27].

## Discussion

Heat shock proteins function in various biological and physiological processes and may be produced in response to temperature, starvation, or disease [33–37]. In this study, we showed that the coding regions of five *L*. *trifolii hsps* are highly conserved relative to that in *L*. *huidobrensis* and *L*. *sativae*. The C-terminal ends of *Lt*HSP90 and *Lt*HSP70 both contain EEVD motifs, which is consistent with their role as molecular chaperones for interaction with other proteins [38]. *Lt*HSP60 also contained C-terminal (GGM)_n_ repeats, which are typical of mitochondrial forms of HSP60 [39]. However, the other *Liriomyza* HSPs are likely located in the cytosol. *Lt*HSP40 contained a DnaJ domain near the N-terminus, which is consistent with its function in ATPase activity and role as a co-chaperone with HSP70 in multiple processes (e.g. protein folding, trafficking, assembly, and dissociation) [40–41]. The central portion of *Lt*HSP20 contained an α-crystalline domain like other sHSPs, may have essential functions in various processes including diapause and insect immunity [42–43].

Although the coding regions of *Liriomyza* species *hsps* are highly conserved, the nucleotide sequences of the 5’UTRs in the *Lthsp* were different from the other two *Liriomyza* spp. Regulatory elements in the *hsp* promoter regions play important roles in *hsp* expression and may be a contributing factor in establishing specific patterns of *hsp* expression [44–45]. Ten TATA-box-like elements were identified in the 5’UTRs of the five *Lthsps*, and the number of 5’UTRs and their locations varied among the three *Liriomyza* spp. (Fig 3). Data on different *T*_on_ and *T*_max_ values of *hsps* expression in the three *Liriomyza* may explain the differences in the ability of three *Liriomyza* species to tolerate cold and heat stress. Although *L*. *huidobren*sis prefers cold climates [46], *L*. *trifolii* may be the most cold-tolerant when compared to the other two *Liriomyza* species based on *hsps* expression. The differences of heat tolerance among the three species were relatively small. *L*. *trifolii* and *L*. *sativae* may have comparative thermotolerance than *L*. *huidobrensis* according to *hsps* expression pattern.

Furthermore, the super cooling points (SCPs) of *L*. *trifolii* and *L*. *huidobrensis* were less than -20°C, which was much lower than that of *L*. *sativae* (-11.7°C) [30, 47–48]. SCP is an important predictor for cold tolerance [49–51] and field populations of *Liriomyza* appear to enhance their cold tolerance by depressing the SCP of the puparial stage [1, 52]. The SCP value for *L*. *trifolii* was low enough to enable the leafminer to safely overwinter in most regions of China. However, *L*. *trifolii* is primarily distributed in southern China, thus further research is needed to discover underlying reasons for its southern distribution patterns. Other studies have shown that the developmental threshold temperature and effective accumulated temperature of the three leafminers were different [53–55]. The developmental threshold temperature of each life stage of *L*. *trifolii* was lower than that of *L*. *sativae*. Therefore, the first generation of *L*. *trifolii* should occur earlier than *L*. *sativae*. In addition, the effective accumulated temperature of *L*. *trifolii* was higher than that of *L*. *sativae*, resulting in the longer dissemination period of *L*. *trifolii* relative to *L*. *sativae*. In contrast, *L*. *huidobrensis* occurs at relatively high latitudes and altitudes. The developmental threshold temperature of *L*. *huidobrensis* was the lowest among the three leafminers, and the effective accumulated temperature was in-between *L*. *sativae* and *L*. *trifolii*. The earlier occurrence of the first generation and longer dissemination in *L*. *trifolii* suggest that this pest could probably expose to cold climates in spring/post-winter and autumn/pre-winter, and its relatively lower cold tolerance may play an important role in the survival and development of the population. [1, 56–57].

Although it has the potential to be widely dispersed throughout the country, *L*. *trifolii* was currently limited to southern China [3, 58]. There may be several reasons for the geographical limitation of *L*. *trifolii*. *Liriomyza sativae* and *L*. *huidobrensis* were first identified in China in 1994 [59–60] while *L*. *trifolii* was first reported eleven years later in 2005. *Liriomyza trifolii* may not have had enough time to disperse into other regions of China. In addition, *L*. *sativae* has a higher reproductive capacity than *L*. *trifolii* [61] and may not be as prolific as *L*. *sativae*. Other possible reasons include the occurrence of natural enemies, pesticide resistance, and the availability of host plants [61–69].

With the rapid development of facility agriculture in China, *L*. *sativae* has been displaced by *L*. *trifolii* in the southern region of the country. In Hainan province, *L*. *trifolii* has become the dominant species, and it constitutes about 95% of the leafminer population in the city of Sanya [70]. Field investigations of *Liriomyza* spp. have revealed that the damage caused by *L*. *trifolii* has become a more serious pest in the northern part of Jiangsu from 2008 to 2015 [71–72]. In the context of predictions of global warming, and the development of facility agriculture and frequent trade exchange, it is high possible that the range of *L*. *trifolii* will expand in China. Therefore, research is critical regarding the factors affecting the distribution of *L*. *trifolii* in China.
